# The DCR Protein TTC3 Affects Differentiation and Golgi Compactness in Neurons through Specific Actin-Regulating Pathways

**DOI:** 10.1371/journal.pone.0093721

**Published:** 2014-04-02

**Authors:** Gaia Elena Berto, Cristina Iobbi, Paola Camera, Elena Scarpa, Corinne Iampietro, Federico Bianchi, Marta Gai, Francesco Sgrò, Flavio Cristofani, Annette Gärtner, Carlos G. Dotti, Ferdinando Di Cunto

**Affiliations:** 1 Department of Molecular Biotechnology and Health Sciences, University of Torino, Torino, Italy; 2 VIB Center for the Biology of Disease – VIB, Leuven, Belgium; 3 Centro de Biología Molecular Severo Ochoa, CSIC/UAM, Madrid, Spain; University of Birmingham, United Kingdom

## Abstract

In neuronal cells, actin remodeling plays a well known role in neurite extension but is also deeply involved in the organization of intracellular structures, such as the Golgi apparatus. However, it is still not very clear which mechanisms may regulate actin dynamics at the different sites. In this report we show that high levels of the TTC3 protein, encoded by one of the genes of the Down Syndrome Critical Region (DCR), prevent neurite extension and disrupt Golgi compactness in differentiating primary neurons. These effects largely depend on the capability of TTC3 to promote actin polymerization through signaling pathways involving RhoA, ROCK, CIT-N and PIIa. However, the functional relationships between these molecules differ significantly if considering the TTC3 activity on neurite extension or on Golgi organization. Finally, our results reveal an unexpected stage-dependent requirement for F-actin in Golgi organization at different stages of neuronal differentiation.

## Introduction

Neuronal cells are characterized by a high degree of morphological and functional polarization, which is required for their activity as information processors and is progressively established during development [1]–[3]. Neuronal polarization, occurring during the earliest stages of differentiation, is driven by the interaction of cell-extrinsic and cell-intrinsic mechanisms, impinging on growth cone cytoskeletal dynamics and on polarized membrane trafficking [4], [5].

During the fast axonal growth phase, which follows neuronal polarization, the Golgi apparatus has a very compact organization and is oriented towards the growing axon [1], [6]. However, since dendrites initiate their rapid growth phase, the Golgi becomes more dispersed and is oriented towards the dendrites [6], which very often contain specialized Golgi outposts associated with branch points [4], [7].

The dynamics of actin cytoskeleton play an essential role at the growth cone [8]–[10] and at the Golgi [11], [12] and are likely to be involved in the crosstalk between these two structures during neurite extension. Remodeling of F-actin at the growth cone is essential for neuronal polarization, since the neurite which will be specified as the axon is characterized by increased actin instability [13]. This condition allows the penetration of microtubules, which can direct membrane flow from the Golgi to the growing tip [8], [14]. On the other hand, while in most cell types depolymerization of the actin cytoskeleton does not disrupt Golgi compactness [11], it may elicit Golgi fragmentation in differentiating neurons [15].

As in all the other cell types, cytoskeletal dynamics of neuronal cells are primarily orchestrated by small GTPases of the Rho family and by their effector networks [2]. Rho-GTPases may act both at the growth cone and at the Golgi, strongly suggesting that they could mediate the dynamic crosstalk between these structures during differentiation.

This possibility is strongly supported in the case of Cdc42 and of its effectors. Indeed, Cdc42 is enriched in the Golgi [16] and is accumulated by polarity cues at the axonal growth cone, where it may promote actin instability and actin retrograde flow by increasing the local phosphorylation of Cofilin, which is mediated by the ser/thr kinase LIM-kinase [17]–[21]. This can be in turn activated by the Rac1/Cdc42 effectors PAK kinases [22] or by the RhoA effectors Rho-kinases (ROCKs) [23]. In addition, in neuronal cells, LIMK can promote the delivery of the PAR3/PAR6 polarity complex to the growth cone [21]. Together, these reports suggest that the CDC42 to LIMK pathway may sustain a positive feedback loop between the Golgi and the growth cone, which may maintain polarity. Accordingly, the CDC42 effectors PAK4 [18] and LIMK1 [21] are localized both at the growth cone and at the Golgi; in the case of LIMK1 it has also been shown that Golgi localization is important for axon outgrowth [21].

Previous studies suggest that also RhoA could mediate a Golgi to growth cone crosstalk. It is well established that RhoA inhibits growth cones extension through a pathway connecting RhoA with the neuro-specific Profilin IIa isoform (PIIa) [24] through ROCKs [2], [10]. In addition, we found that active RhoA is specifically associated with the Golgi of differentiating neurons [15] and that it may regulate Golgi compactness through Citron-N (CIT-N) [25], a CNS-specific variant of the cytokinesis regulator Citron-kinase (CIT-K) [26]–[28]. Since CIT-K is capable to inhibit neuronal differentiation [29], it is conceivable that it may share this activity with CIT-N, but this has not been demonstrated directly so far.

The product of the TTC3 gene [30] is an interesting possible regulator of these molecules in neuronal differentiation. TTC3 is one of the genes mapped to the Down Critical Region, a relatively small locus of human Chr. 21 that plays a major role in generating the characteristic intellectual disability of Down syndrome [30]. The encoded protein contains four TPR motifs in the amino-terminal half, a potentials coiled-coil region and a Citron binding region in the central part and a E3 ubiquitin ligase ring-finger domain at the carboxi terminus [31], [32]. The only substrate of the ubiquitin-ligase activity of TTC3 so far reported is the phosphorylated form of Akt [33]. Expression profiling studies, performed in human cells from patients and in mouse models have shown that TTC3 is one of the most consistently upregulated genes in a Chr. 21-related trisomic context and in other disorders associated with intellectual disability [34]. These data suggest that increased expression levels of TTC3 in neuronal cells may significantly contribute to the phenotypes that characterize Down syndrome (DS).

In agreement with this possibility, we previously found that TTC3 inhibits the neuronal-like differentiation of pheocromocytoma cells by activating RhoA and by binding to Citron proteins [32]. However, TTC3 has never been studied so far in primary neuronal cells. Moreover, it is not known whether, being a Citron partner, TTC3 may also control neuronal Golgi organization and whether this may relate to its effects on differentiation.

In this report we have used hippocampal neurons in primary culture to investigate the effects of TTC3 levels on neurite extension and Golgi organization during the early stages of neuronal differentiation. Moreover, we analyzed the role of F-actin and the pathways that could connect TTC3 to actin remodeling, using overexpression, RNAi-mediated knockdown or pharmacological inhibition of the critical genes. The results of these experiments and their possible implications are described and discussed.

## Results

### TTC3 Restrains Neuronal Differentiation by Modulating F-actin Stability: Role of CIT-N and of the Other RhoA Downstream Players

**Figure 1:**
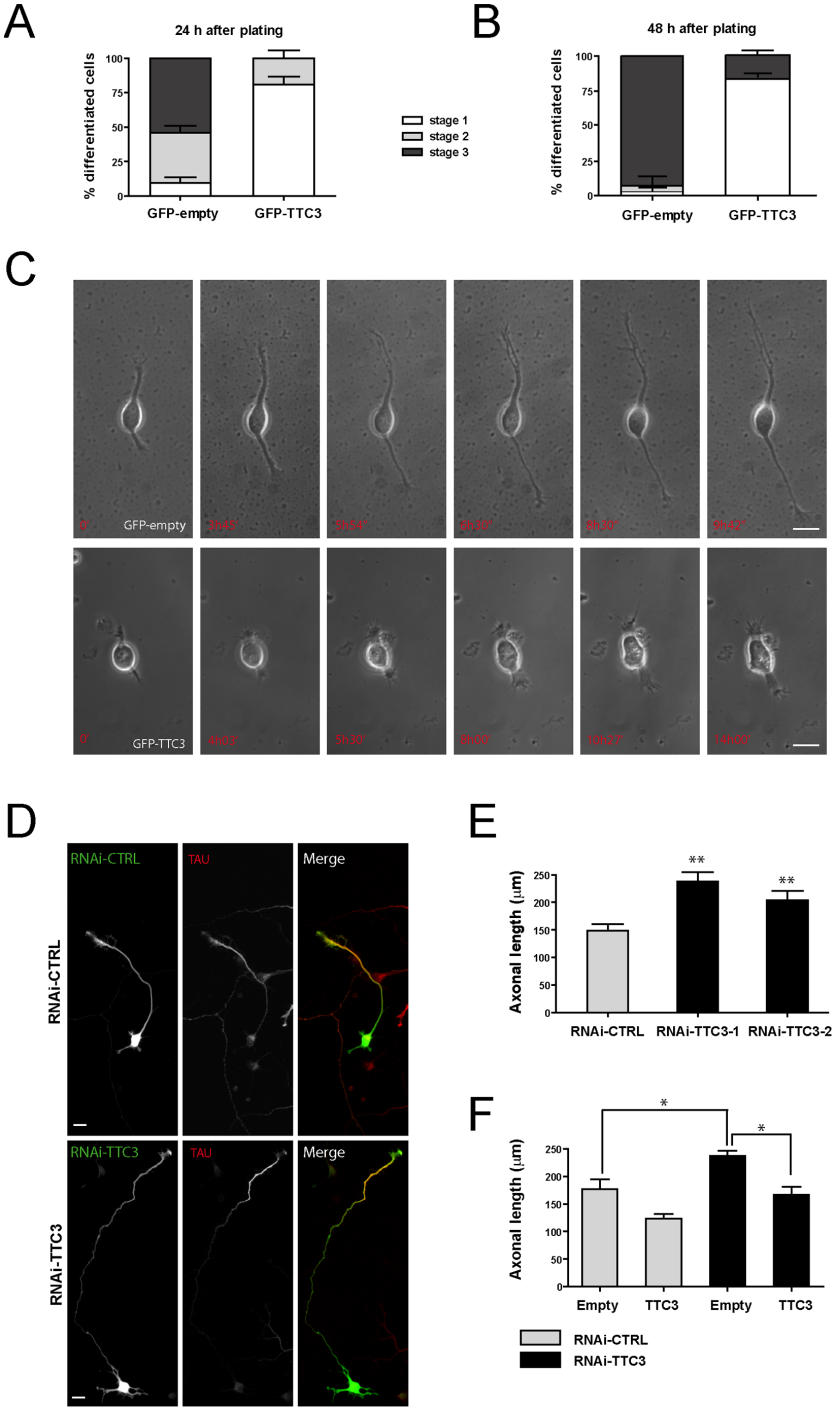
Effects of altered TTC3 levels on neuronal differentiation. **A–B.** Hippocampal neurons extracted from E18.5 rats were electroporated by nucleofection with the indicated plasmids. 24 hours (A) or 48 hours (B) after plating, cells were processed for immunofluorescence and the morphology of GFP-positive cells was assessed to analyze their distribution across the indicated differentiation stages. **C.** Selected frames from time-lapse series of neurons transfected as in panel A, undergoing differentiation in culture. For full movies, see supporting material. **D–E.** Hippocampal neurons were nucleofected with control or TTC3-specific sh-RNA-expressing plasmids, plated and allowed to differentiate 72 hours in culture. Cells were then processed for IF with anti-Tau antibodies and the axonal length was then quantified with ImageJ. **F.** Overexpression of GFP-TTC3 (TTC3) is able to rescue the phenotype induced by TTC3 downregulation. Cells were co-electroporated with sh-RNA-expressing plasmids together with GFP-empty (empty) or GFP-TTC3 (TTC3). After 72h, cells were than analyzed as in panel E. Scale bars = 10 μm; error bars = Standard Error of the Mean (SEM); *P<0.05, **P<0.01, two tails Student T-test.

To address whether TTC3 can modulate the physiologic neuronal differentiation program, we resorted to rat hippocampal neurons in primary culture [35], which were first used to perform overexpression studies. In particular, neurons were electroporated immediately before plating with plasmids expressing GFP-TTC3 from a CMV promoter and their morphology was assessed 24 and 48 hours after transfection. The typical transfection efficiency was around 30% and transfected cells displayed TTC3 levels 5 to 10 folds higher than endogenous levels (Fig. S1A). TTC3 overexpression strongly impaired neuronal differentiation, with only 20% of the neurons displaying a multi-neurite morphology (stage 2, [35]) at 24 hours (Fig. 1A), or displaying a clearly polarized morphology (stage 3, [35]) at 48 hours (Fig. 1B). In comparison, 50% and 90% of cells transfected with a control GFP plasmid reached Stage 3 at 24 hours and 48 hours, respectively (Fig. 1A–B). Time-lapse recording (Fig. 1C, Movies S1 and S2) showed that cells expressing high TTC3 levels are capable to progress from spherical morphology (stage 1, [35]) to a bipolar shape, but are then defective in neurite elongation. To establish whether the over-expression paradigm reflects the role of TTC3 in the physiological context, i.e. it is a physiological modulator of neurite outgrowth, we set out to reduce its expression by RNA interference, using two independent sequences which have been previously validated [32]. Primary neurons were transfected in suspension shortly after dissection and analyzed 72 hours later. The typical transfection efficiency was around 40% and shRNA-expressing constructs reduced TTC3 immunoreactivity at levels of approximately 30%, if compared to controls (Fig. S1B). Axonal length was significantly increased in TTC3-depleted neurons (Fig. 1D–E). As expected, this phenotype was reverted by cotransfection of an expression construct encoding full length TTC3 (Fig. 1F). Since axon is the neurite growing at higher speed in this window of the differentiation program, these results are consistent with the possibility that TTC3 plays a physiological role in neurite growth.

**Figure 2:**
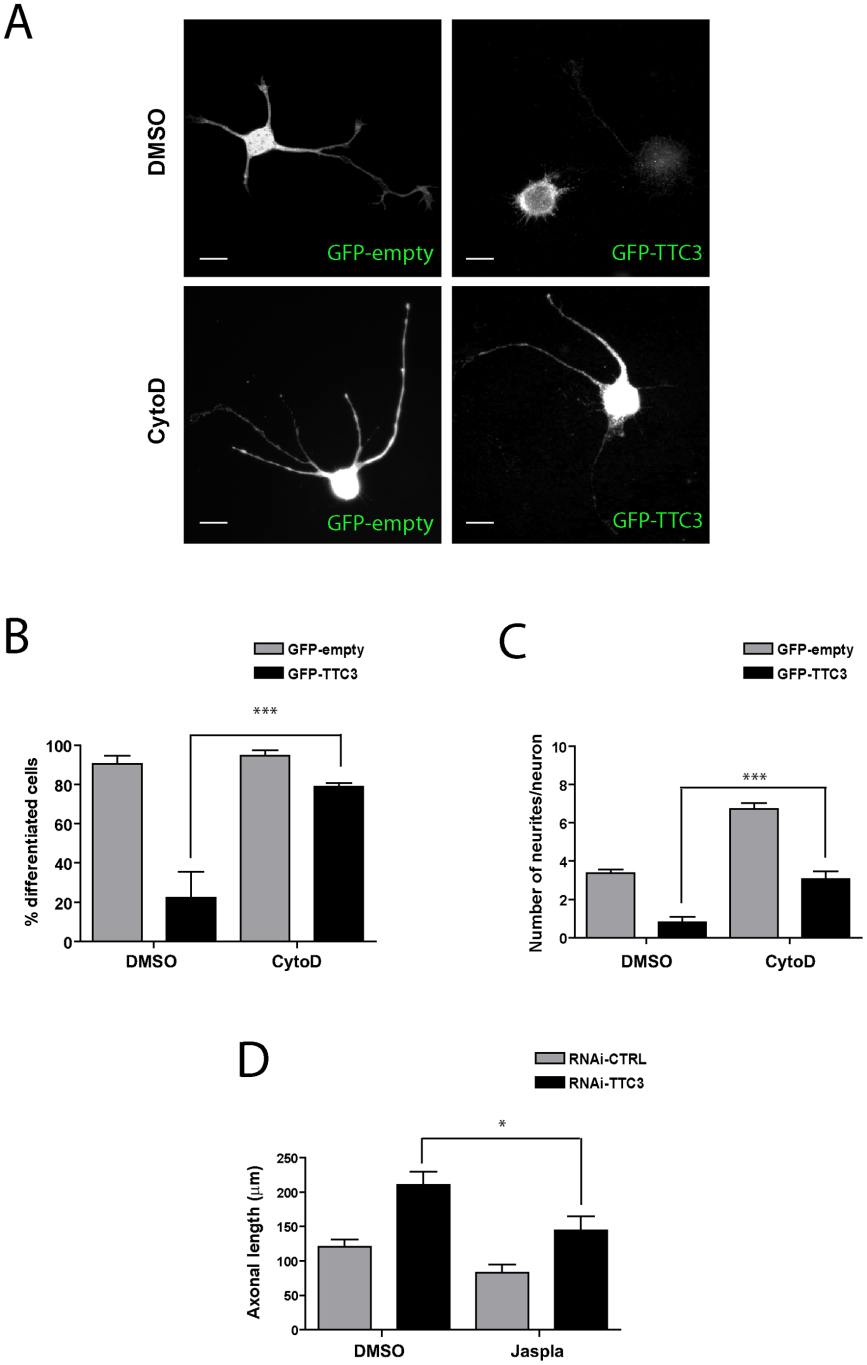
Effects of actin-affecting drugs on the neurite-extension phenotypes induced by modulating TTC3 levels. **A.** Hippocampal neurons were electroporated by nucleofection with the indicated plasmids and treated after 6 hours with vehicle (DMSO) or with 1 μM Cytochalasin-D (CytoD). 18 hours later cells were then processed for IF. Neurites were revealed by the GFP signal. **B.** Quantification of the percentage of differentiated cells in hippocampal neurons treated as in panel A. Differentiated cells were defined as those bearing at least one neurite longer than twice the cell body. **C.** Quantification of the average number of neurites in hippocampal neurons treated as in panel A. **D.** Hippocampal neurons were nucleofected with control or with TTC3-specific sh-RNA-expressing plasmids, plated and allowed to differentiate 54 hours in culture. Cells were then treated with 5 nM Jasplakinolide (Jaspla) or with vehicle for additional 18 hours and processed for IF to reveal GFP and Tau. The length of the main neurite (Tau-positive axon) was then quantified. Scale bars = 10 μm; error bars = SEM; *P<0.05, ***P<0.001, two tails Student T-test.

To address the role of the actin cytoskeleton in the phenotype induced by abnormal TTC3 levels, we tested whether these can be modified by changes of the polymerization state of actin. In particular, since the overexpression of TTC3 can be detected already 6 hours after transfection (data not shown), to evaluate the role of actin polymerization on the overexpression phenotype we measured the percentage of differentiated cells (i.e. of cells possessing at least one neurite longer than twice the cell body) and the average number of neurites per cell, which are two fundamental parameters of the early differentiation stages. Moreover, since in RNAi experiments the reduction of TTC3 can be detected only 72 hours after transfection, to evaluate the role of actin polymerization on the knockdown phenotype we measured axonal length. Interestingly, in TTC3 overexpressing neurons, the percentage of differentiated cells (Fig. 2A–B) and the number of neurites (Fig. 2C) were restored to control values by treatment with 1 μM of the actin-destabilizing drug Cytochalasin D (CytoD). Conversely, Jasplakinolide (Jaspla), a macrocyclic peptide which stimulates actin nucleation and induces actin polymerization, reverted to control levels the increased axonal length induced by TTC3 knockdown (Fig. 2D).

**Figure 3:**
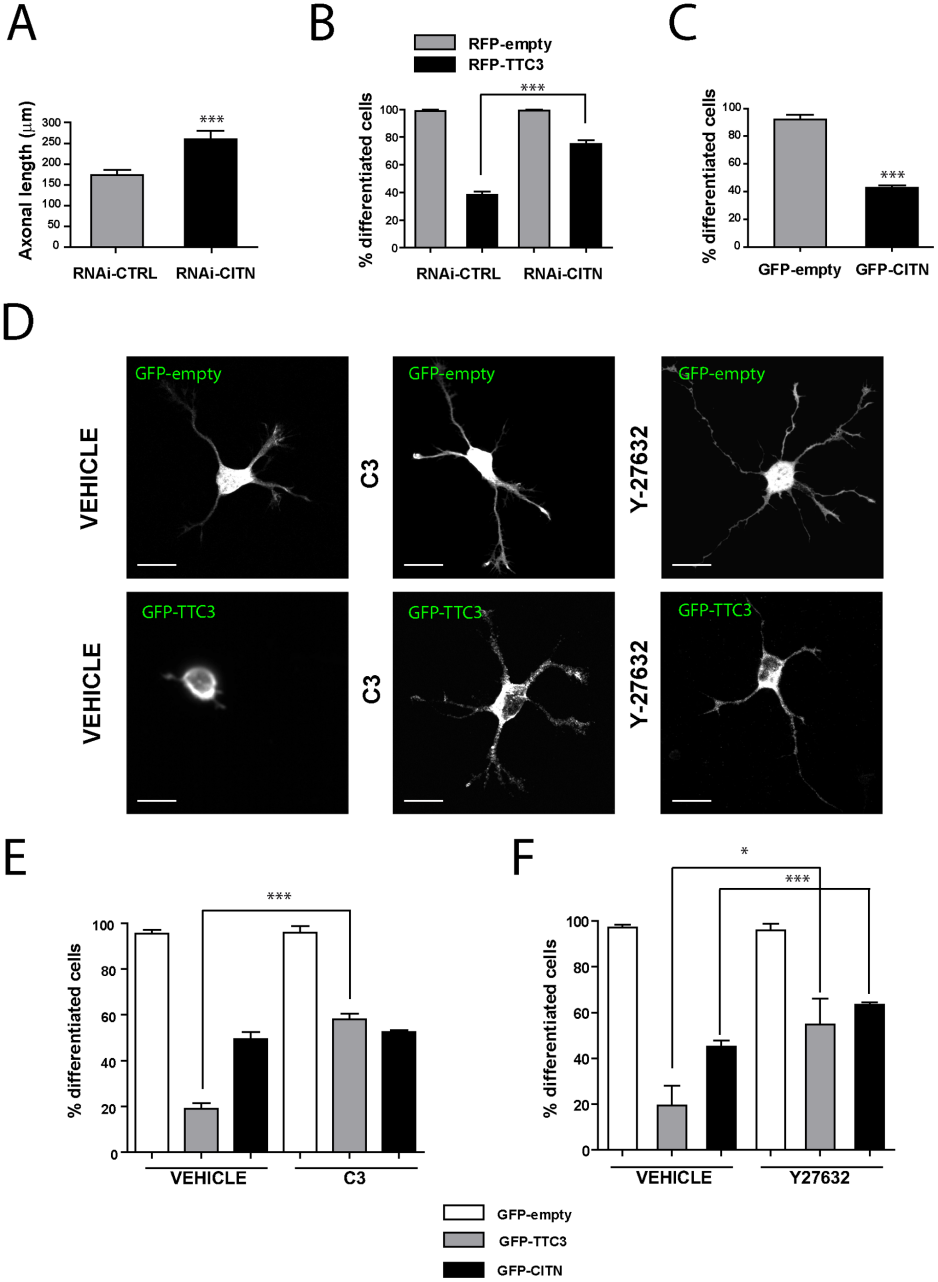
Role of TTC3 partners in TTC3-dependent neurite extension phenotypes. **A.** Hippocampal neurons were nucleofected with control or CIT-N-specific sh-RNA-expressing plasmids, plated and allowed to differentiate 72 hours in culture. Cells were then processed for IF with anti-Tau antibodies and the axonal length was quantified. **B.** Hippocampal neurons were nucleofected with control or CIT-N-specific sh-RNA-expressing plasmids, together with plasmids overexpressing, under a CMV promoter, RFP or RFP-TTC3, as indicated. The percentage of differentiated cells was then assessed as in the previous figures. **C.** Hippocampal neurons were electroporated by nucleofection with the indicated plasmids. 24 hours after plating cells were processed for immunofluorescence and the morphology of GFP-positive cells was assessed to analyze the percentage of differentiated cells. **D–F.** Hippocampal neurons were nucleofected with the indicated plasmids, allowed to differentiate in culture for 24 hours and treated with vehicle, with cell permeable C3 toxin at the final concentration of 2 μg/ml during the last 4 hours or with the ROCK inhibitor 36,7 μM Y27632 during the last 18 hours. Neurites were revealed by the GFP signal. The percentage of differentiated cells (E) was then assessed as in the previous figures. Scale bars = 10 μm; error bars = SEM; *P<0.05, ***P<0.001, two tails Student T-test.

We previously found that in PC12 cells the excess of TTC3 inhibits differentiation by activating RhoA, through a pathway requiring CIT-K but not impinging on Rho kinases (ROCKs) [32]. Although it appears reasonable to postulate that a similar mechanism may operate in neurons, it is also possible that TTC3-dependent events occurring in primary neurons are significantly different from those characterizing immortalized neuroendocrine cell lines. For instance, an important difference is that post-mitotic neurons express CIT-N but do not express CIT-K [15], [28], [36], while PC12 and neuroblastoma cells express only CIT-K regardless of their differentiation state [29], [32]. Therefore, we assessed the role of CIT-N, RhoA and ROCK in the events activated by TTC3 overexpression in hippocampal neurons during the early stages of differentiation. We first asked whether, as in the case of CIT-K in neuroblastoma cells [29], CIT-N may restrain neurite outgrowth in primary neurons. This was indeed the case, since the knockdown of CIT-N increased axon outgrowth in primary neurons (Fig. 3A) similarly to TTC3 knockdown. Moreover, CIT-N knockdown significantly increased the number of differentiated cells in TTC3-overexpressing neurons (Fig. 3B), thus indicating that CIT-N is a physiological modulator of neuronal differentiation acting downstream of TTC3. Consistent with this observation, the overexpression of CIT-N significantly reduced the percentage of differentiated neurons (Fig. 3C). Inhibition of RhoA in TTC3-overexpressing neurons, obtained through a cell-permeable C3 toxin, significantly increased the number of differentiated cells (Fig. 3D–E), a result consistent with the previous findings [32]. However, in contrast with the results obtained in PC12 cells [32], inhibition of ROCK by the Y27632 compound partially rescued the differentiation phenotype (Fig. 3D and 3F), thus indicating that in primary neurons ROCK acts downstream of TTC3. Inhibition of RhoA did not rescue the differentiation phenotype induced by CIT-N overexpression (Fig. 3E), suggesting that in this context CIT-N acts downstream of RhoA. Moreover, the inhibition of ROCK in CIT-N overexpressing neurons significantly increased the percentage of differentiated cells (Fig. 3F), indicating that ROCK may act downstream of CIT-N. Taken together, these results provide evidence that TTC3 levels affect neuronal differentiation at least in part through actin remodelling via RhoA, CIT-N and ROCK, with these molecules acting in a linear pathway.

### Increased Levels of TTC3 Disrupt Neuronal Golgi: Role of CIT-N and of the other RhoA Downstream Players

**Figure 4:**
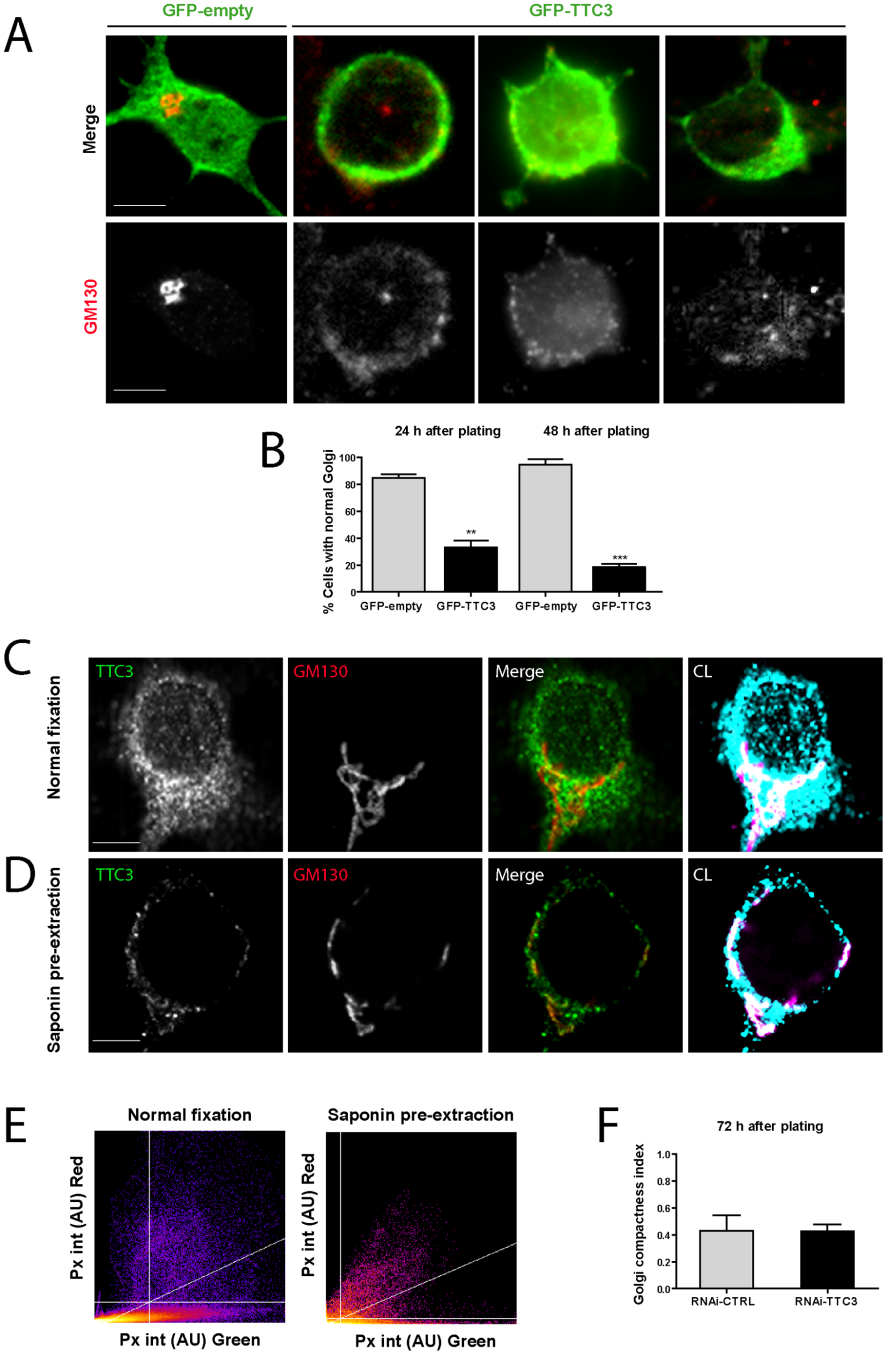
Effects of altered TTC3 levels on Golgi compactness. **A–B.** Hippocampal neurons were electroporated by nucleofection with the indicated plasmids. Cells were processed for immunofluorescence with anti-GM130 antibodies 24 or 48 hours after plating. The percentage of cells with compact (normal) or fragmented Golgi morphology was then quantified (B). Note the compact morphology of the Golgi in the cell transfected with the GFP control plasmid (first cell from left in panel A). The other three cells in panel A are different examples of TTC3-overexpressing cells with disrupted Golgi, analyzed 24 hours after transfection. **C–D.** Confocal images of 7 DIV primary hippocampal neurons co-stained for TTC3 and GM130 after standard PFA fixation (C) or after pre-extraction with saponin (D). The right panels show a false color imange in which the white pixels represent the areas of colocalization (CL) of TTC3 and GM130 (cyan and magenta, respectively). **E.** Pixel intensity plots of the TTC3 and GM130 immunoreactivities in exemplar cells treated as in panels C and D. AU = arbitrary units. **F.** Hippocampal neurons were nucleofected with control or TTC3-specific sh-RNA-expressing plasmids (RNAi-TTC3), plated and allowed to differentiate 72 hours in culture. Cells were then processed for IF with GM130 antibodies and the Golgi compactness was measured (see Material and methods). Scale bars = 5 μm; error bars = SEM; **P<0.01, ***P<0.001, two tails Student T-test.

Because of the close functional relationship between morphological differentiation and organelle polarization [37]–[39], of the previously reported interaction between Citron proteins and TTC3 [32], and of the involvement of CIT-N in neuronal Golgi organization during the late stages of neuronal differentiation [15] we investigated whether the excess or paucity of TTC3 and CIT-N affect Golgi organization at early differentiation stages. The overexpression of TTC3 had a dramatic effect, since most of the TTC3-overexpressing cells displayed a fragmented Golgi already 24 hours after transfection (Fig. 4A–B). This phenotype led us to analyze by immunofluorescence whether TTC3 is localized to this organelle in physiological conditions. Under normal fixation, TTC3 is diffusely detectable in the cytoplasm and in the neurites of primary neurons, in which it displays a polarized enrichment (Fig. S1B and 4C). Interestingly, in cells in which the cytosolic pool of TTC3 was extracted with saponin before fixation, most of the TTC3 immunoreactivity was eliminated and the residual signal showed a clear colocaIization with Golgi markers (Fig. 4D). The good colocalization was confirmed by a pixel-by-pixel analysis (Fig. 4E). This revealed that the percentage of TTC3-positive GM130 pixels is close to 100% in both cases, while the percentage of GM130-positive TTC3 pixels is approximately three times higher in pre-extracted cells (Fig. 4E). Under the same conditions, no enrichment at the growth cone could be detected (data not shown). We then asked whether TTC3 is a physiological determinant of Golgi compactness, by performing RNAi as described above. Since no gross qualitative effects could be detected by expert inspection, we performed a quantitative analysis of Golgi compactness in TTC3-dpeleted cells, using a previously described strategy [40]. However, even in this case no differences with control cells could be detected (Fig. 4F).

**Figure 5:**
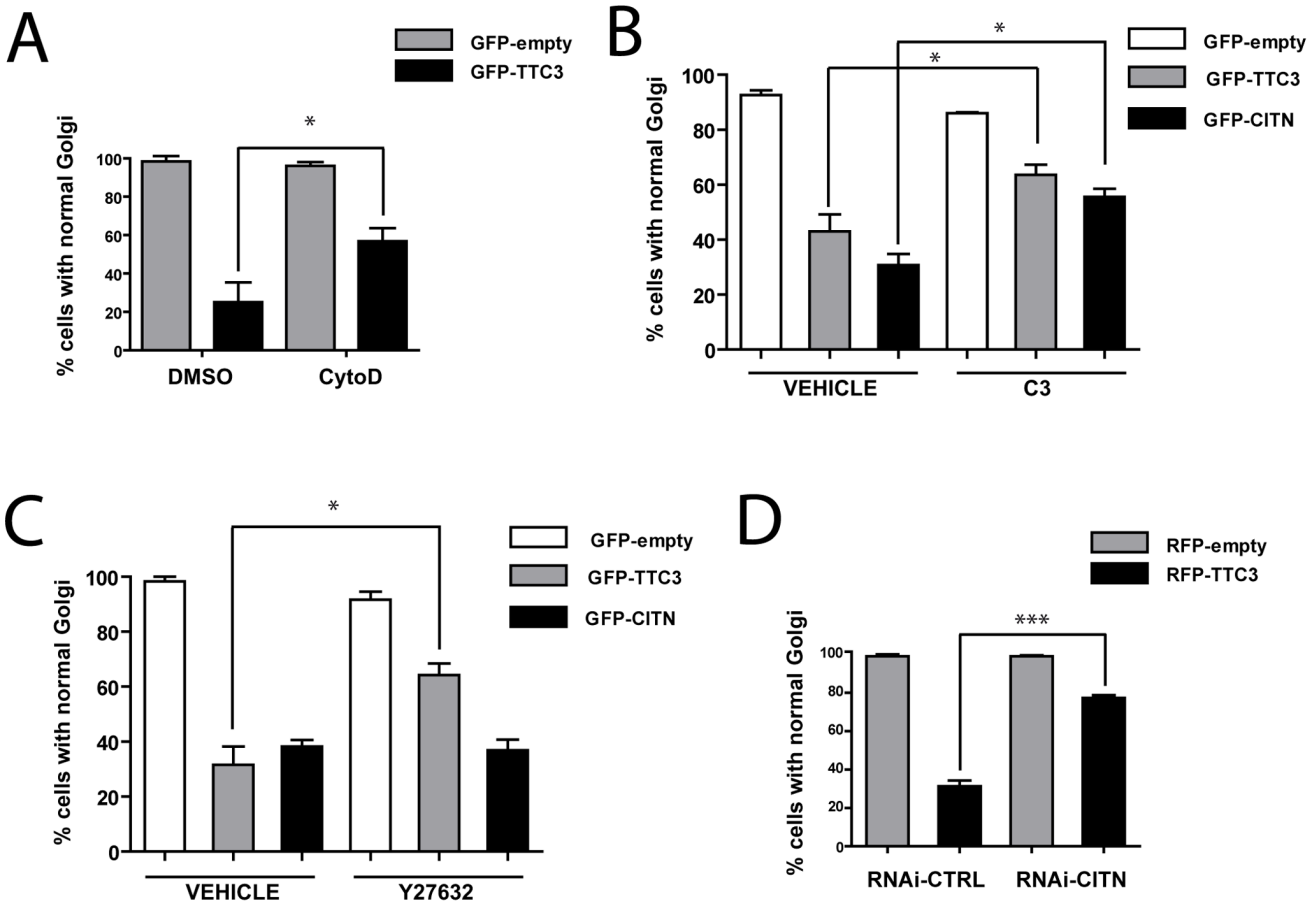
Role of TTC3 partners in TTC3-dependent Golgi fragmentation. **A.** Hippocampal neurons were electroporated by nucleofection with the indicated plasmids and treated with vehicle (DMSO) or with 1 μM Cytochalasin-D (CytoD) for an additional 24 hours. Cells were then processed for IF with the Golgi GM130 marker and quantified for normal Golgi as in Fig. 4. **B–C.** Hippocampal neurons were nucleofected with the indicated plasmids, allowed to differentiate in culture for 24 hours and treated as described in Fig. 2 with vehicle, C3 toxin and Y27632. Vehicle was 50% glycerol in the case of C3 and water in the case of Y27632. Cells where then processed and analyzed as in panel A. **D.** Hippocampal neurons were nucleofected with control or CIT-N-specific sh-RNA-expressing plasmids (RNAi), together with plasmids overexpressing, under a CMV promoter, RFP or RFP-TTC3, as indicated. The percentage of cells with normal Golgi was then assessed as in the previous panels. Scale bars = 10 μm; error bars = SEM; *P<0.05, ***P<0.001, two tails Student T-test.

These results indicate that TTC3 can associate with Golgi membranes and that abnormal TTC3 levels can alter Golgi organization, although TTC3 is most likely not involved in regulating basal Golgi structure. We then assessed whether, as in the case of neurite extension, the effects of TTC3 overexpression on Golgi are mediated by increased actin polymerization. Treatment with CytoD decreased significantly the number of TTC3-overexpressing neurons with fragmented Golgi, even though this was not reverted to control values (Fig. 5A). This result was not expected since we previously found that CytoD induces Golgi fragmentation in neuronal cells [15]. However, the fact that the previous experiments were performed at 7 DIV, while the present experiments were performed at 1 DIV raised the possibility that the status of actin cytoskeleton may affect Golgi organization in different manner at different stages of neuronal development. We therefore checked the effects of 1 μM and of 2.5 μM CytoD on our cultures at 1 DIV and at 7 DIV. We thus confirmed that CytoD has no effect on Golgi compactness at 1 DIV, while it induces Golgi fragmentation at 7 DIV (Fig. S2).

Next, we analyzed the role RhoA, ROCK and CIT-N in the TTC3-induced Golgi fragmentation phenotype. Inhibition of RhoA and of ROCK partially restored Golgi compactness (Fig. 5B and 5C), with quantitatively similar effects. Moreover, we found that also CIT-N knockdown was capable to partially rescue the Golgi fragmentation phenotype induced by TTC3 overexpression (Fig. 5D). This result was unexpected since we previously found that, at 7 DIV, CIT-N is required to maintain Golgi compactness [15]. It suggests that, as in the case of actin, also the requirement for CIT-N during the first stages of differentiation could be significantly different if compared to later time points. Accordingly, we found that CIT-N depletion in 1 DIV cells does not affect Golgi compactness (Fig. 5D). In addition, the overexpression of CIT-N at 1 DIV induced a Golgi fragmentation phenotype comparable with the phenotype induced by TTC3 overexpression (Fig. 5B–C). Even in this case the phenotype was partially rescued by RhoA inactivation (Fig. 5B). In contrast, the Golgi fragmentation induced by CIT-N overexpression was not modified by ROCK inhibition (Fig. 5C). These results indicate that abnormally high levels of TTC3 alter Golgi organization, at least in part through actin hyperpolymerization mediated by RhoA, CIT-N and ROCK. However, the relationships between CIT-N and the other two players are significantly different if compared to neurite extension phenotype (see discussion).

### The Neuronal Phenotypes Induced by TTC3 Overexpression are Reverted by Increased LIMK and Decreased PIIa Levels

**Figure 6:**
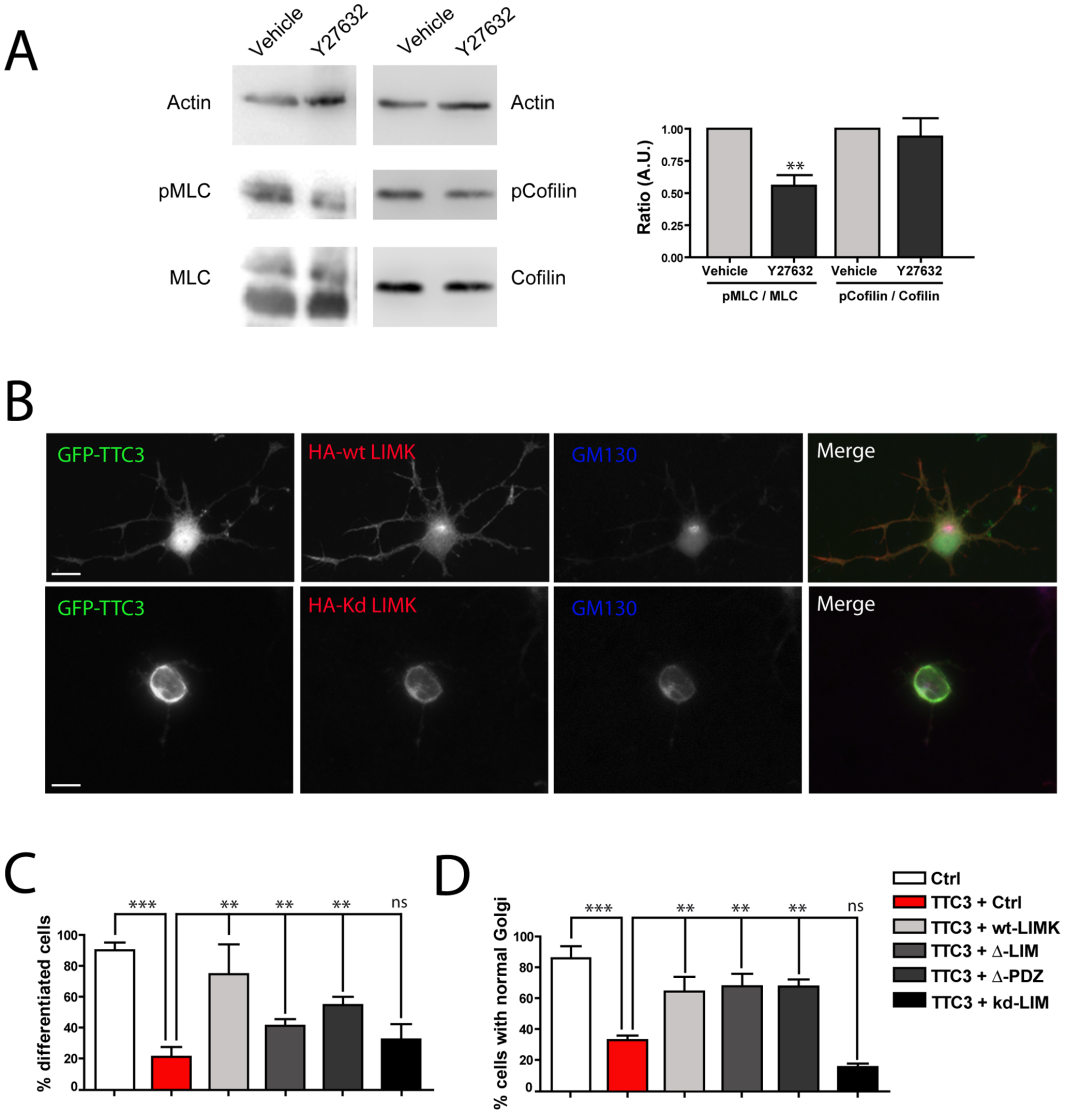
Analysis of the role of LIMK in TTC3-dependent phenotypes. **A.** 1 DIV primary hippocampal neurons plated on biochemistry dishes were treated for 24h with vehicle or with the ROCK inhibitor Y27632. Cells were then lysed and the expression of the indicated proteins was analyzed by western blotting with the indicated antibodies. In the left panel the ratio between phopshorylated and non-phosporylated forms of myosin light chain (MLC, positive control) and of Cofilin was quantified. pMLC and pCofilin indicate the phosphorylated forms of the two proteins. The result represents the average of three independent experiments. **B–D.** Primary hippocampal neurons were co-transfected with the CMV expression plasmids encoding GFP-TTC3 and with HA empty plasmid (Ctrl), HA tagged wild type LIMK (wt) or with the indicated HA-tagged LIMK mutants. Cells were then processed for IF to reveal the expression of the encoded proteins and with anti-GM130 antibodies. The percentage of differentiated cells (C) and of cells with intact Golgi (D) was then quantified. Scale bars = 10 μm; error bars = SEM; **P<0.01, ***P<0.001, ns = non significant, two tails Student T-test.

Based on current models, the RhoA effector ROCK could increase actin polymerization downstream of TTC3 by two different pathways. On one hand, ROCK may increase the amount of F-actin through the activating phosphorylation on Thr-508 of LIMK [23], which in turn inhibits the actin-depolymerizing protein Cofilin by phosphorylating its Ser3 residue [17]. This pathway would be particularly attractive to explain the phenotypes described above, because LIMK can localize both at the growth cone and at the Golgi, through its PDZ and LIM domains, respectively [21]. However, it is still not clear whether this pathway actually operates in differentiating neurons at early times. For instance, the same activating phosphorylation on LIMK can be produced by PAK kinases, operating downstream of Rac and Cdc42 [22]. To address whether the ROCK/LIMK/Cofilin pathway is actually working during the early stages of neuronal differentiation, we analyzed the effects of ROCK inhibition on Cofilin phospholrylation. We found that the ratio between Ser3-Cofilin and total Cofilin was not decreased in 1DIV primary neuronal cultures upon ROCK inhibition (Fig. 6A). Moreover, the overexpression of LIMK did not worsen, but rather rescued the phenotypes induced by TTC3 overexpression, both on neurite outgrowth and on Golgi (Fig. 6B–D). LIMK mutants lacking either the PDZ or the LIM domains were capable to partially rescue the effects of TTC3 on neurite outgrowth and to totally rescue the Golgi phenotype (Fig. 6C–D). In contrast, a kinase dead LIMK mutant did not affect both TTC3-induced phenotypes (Fig. 6C–D). These results argue against a role of LIMK as a canonical downstream player of ROCK in TTC3 induced phenotypes.

**Figure 7:**
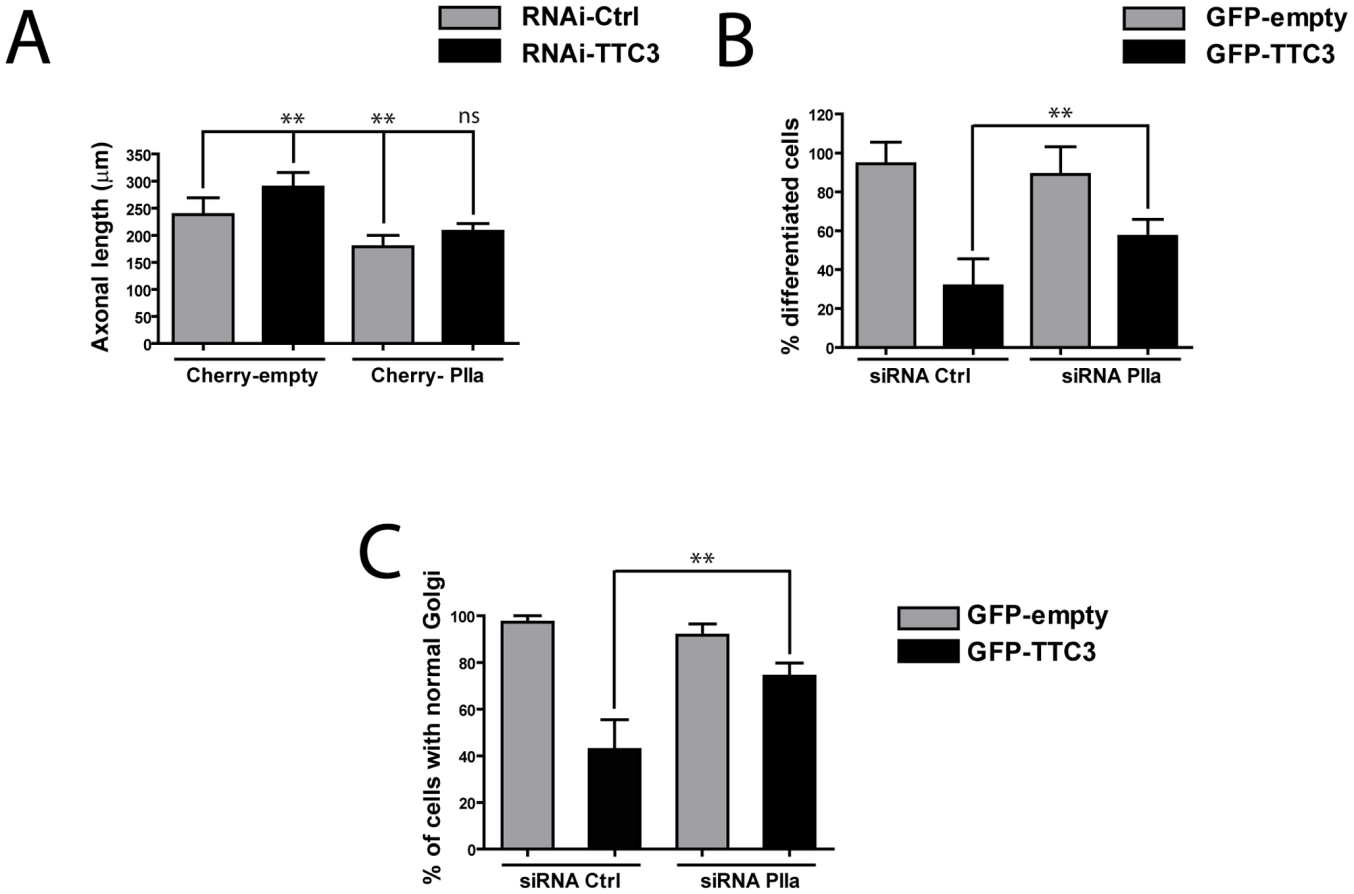
Analysis of the role of PIIa in TTC3-dependent phenotypes. **A.** Hippocampal neurons were nucleofected with control or TTC3-specific sh-RNA-expressing plasmids, together with plasmids overexpressing, mCherry or mCherry-PIIa. Axonal length was then quantified as described above. **B–C.** Hippocampal neurons were nucleofected with control or PIIa-specific sh-RNA-expressing plasmids, together with plasmids overexpressing GFP or GFP-TTC3, as indicated. The percentage of differentiated cells (B) and of cells with normal Golgi (C) was then assessed as in the previous figures. Scale bars = 10 μm; error bars = SEM; **P<0.01, ***P<0.001, two tails Student T-test.

On the other hand, ROCK could increase actin polymerization rate through Profilin IIa (PIIa) [24], a brain-specific form of Profilin playing a fundamental role in neuronal differentiation [2], [41]. Consistent with this possibility, PIIa overexpression reverted neurite length in TTC3-depleted cells to control values (Fig. 7A). Moreover, PIIa inactivation increased significantly the number of differentiated cells (Fig. 7B) and the number of cells with intact Golgi (Fig. 7C) in TTC3-overexpressing cultures, both to levels comparable with those produced by RhoA and ROCK inactivation. These results strongly suggest that TTC3 may affect neurite extension and Golgi organization by increasing actin polymerization through PIIa rather than decreasing actin depolymerization through LIMK and Cofilin.

## Discussion

In this work we have investigated the role of TTC3 in primary neuronal cells during the first stages of differentiation, with respect to both neurite extension and Golgi organization. Moreover, we have addressed the role of some of the possible downstream players which could connect TTC3 to actin organization, especially the CIT-N protein. First of all we have confirmed that, in line with previous studies performed in pheocromocytoma cells [32], TTC3 is a physiological determinant of the neuronal differentiation cell-autonomous program, since its knockdown leads to increased neurite extension. In contrast, the reduction of TTC3 levels is not sufficient in differentiating neurons to alter Golgi organization. Together with the observation that only a minimal fraction of TTC3 is associated with the Golgi, this result suggests that the primary function of TTC3 could be to restrain neurite outgrowth by acting on the growth cone, rather than on the Golgi. However, if TTC3 is expressed above physiological levels, as it may occur in Down syndrome (DS) and other neurological diseases [34], besides impairing neurite extension it can also disrupt Golgi compactness. These findings could be of relevance for understanding how increased levels of TTC3 in DS may contribute to the overall intellectual disability phenotype. Indeed, it is commonly believed that the complex pathological scenario characterizing DS arises from a combination of neurodevelopmental abnormalities and neurodegenerative processes [42]–[44].

Addressing which processes are irreversible and which ones can be prevented or reverted by manipulating genes and pathways is of paramount importance for the possible development of new therapeutic strategies. Impaired neuronal differentiation has been reported by some studies in DS patients [45], [46]. In addition, a prominent role in neuronal differentiation has also been reported for Dyrk1a [47], a gene mapping into the DCR which is considered one of the major players in DS [48]. On the other hand, although Golgi abnormalities have not been reported so far in DS, they have recently been found in several neurodegenerative disorders [49]–[51]. If deeper studies on patients and/or experimental models should confirm the existence of neurite extension or Golgi organization phenotypes in DS, the TTC3 pathway could become an interesting potential target for experimental manipulation.

The effects of TTC3 on neurite extension are mainly mediated by an increase of actin polymerization. Indeed, the enhanced neurite outgrowth produced by TTC3 knockdown is reverted by F-actin stabilization, while the differentiation block produced by TTC3 overexpression is reverted by F-actin depolymerization. An increase in actin polymerization is also involved in the Golgi fragmentation phenotype produced by TTC3 overexpression. At a first glance, this result was in apparent contrast with our previous discovery that in neuronal cells F-actin is required for Golgi compactness [15], a finding which differs from those obtained in other cell types [11]. However, it raised the interesting possibility that in neurons the requirement of F-actin for the structural integrity of the Golgi may change throughout differentiation. We found that this is indeed the case, because CytoD treatment has no effect on Golgi compactness during the earliest stages of differentiation, while it induces Golgi fragmentation at later stages (Fig. S2). The mechanisms responsible for this switch are presently unknown and may represent an interesting subject for future studies.

The effects of TTC3 overexpression on Golgi compactness cannot be explained only by its activity on actin polymerization, since the rescue of the phenotype elicited by CytoD was much less pronounced than the rescue of the neurite extension phenotype. Since it has previously been shown that TTC3 may also affect the PI3-kinase (PI3K)/Akt pathway by ubiquitinating Akt, thus stimulating Akt degradation [33], one possibility is that TTC3 may affect Golgi compactness through this mechanism. However, this possibility would be in contrast with a recent report showing that, in polarized migrating cells, the PI3K/Akt/mTOR pathway controls the polarization of the plasma membrane but does not affect Golgi polarization [52]. It is not known whether these conclusions can also be applied to differentiating neurons. An alternative possibility is that TTC3 may affect Golgi organization by inducing the ubiquitilation of presently unknown substrates. To this regard, it must be noticed that CIT-K was excluded as a possible target by previous studies [33]. On this basis and considering the positive functional relationship between TTC3 and CIT-N we consider very unlikely the possibility that CIT-N may be a TTC3 substrate in neurons.

**Figure 8:**
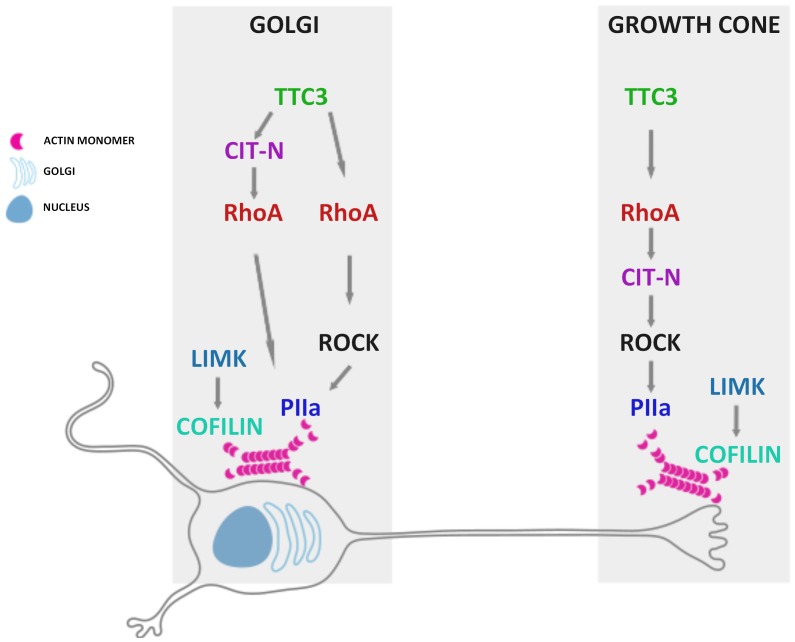
Schematic representation of the TTC3 downstream pathways dissected in hippocampal neurons at the early stages of differentiation in primary culture.

Our results suggest that the molecular networks mediating the effects of enhanced TTC3 expression at the growth cone and at the Golgi may differ significantly, a scenario that was confirmed by our epistatic analysis of TTC3 downstream players (for a summary, see the scheme in Fig. 8). For what concerns neurite extension, our previous studies on pheocromocytoma cells had suggested that TTC3 may limit neurite extension by activating RhoA, which in turn may signal to cytoskeleton through ROCK and CIT-K, with the latter two molecules working in parallel [32]. We have found that the same players operate also in differentiating primary neurons, although in this case Citron proteins are represented by CIT-N. Indeed, the inhibition of RhoA and ROCK and the knockdown of CIT-N are all capable to rescue the neurite outgrowth phenotype induced by TTC3 overexpression. However in this case ROCK regulates neurite extension acting downstream of CIT-N, rather than in parallel with it. Interestingly, the same players are also capable to mediate the Golgi disorganization induced by TTC3 overexpression, but there are at least two important differences with neurite outgrowth inhibition. The first difference is that in this context ROCK does not work downstream of CIT-N. The second is that CIT-N works largely upstream of RhoA, rather than downstream of it. The latter finding is consistent with the data obtained in other biological processes implicating Citron proteins. Indeed, CIT-K and CIT-N have previously been shown to regulate midbody abscission [53], [54] and dendritic spine maintenance [55], [56], respectively, by locally stabilizing active RhoA. To summarize, our data reveal that TTC3 overexpression may affect Golgi organization through at least two pathways (Fig. 8): a “canonical” pathway flowing from RhoA to actin polymerization through ROCK and PIIa; a non canonical pathway involving CIT-N and RhoA, which may impinge on other mechanisms. To this regard, it must be noticed that Citron proteins have recently been linked to microtubules [57], which are well known for their critical role in Golgi organization [58].

Although ROCK is only partially responsible for the effects induced by TTC3 overexpression, it was important to understand how it may affect actin polymerization downstream of TTC3. The reason of this is that ROCK inhibitors display neuroprotective effects in models of neurodegenerative disorders and have already been approved for clinical use [59]. Based on current models, a first mechanism by which ROCK may increase the amount of F-actin is through the activating phosphorylation on Thr-508 of LIMK [23], which in turn inhibits the actin-depolymerizing protein Cofilin by phosphorylating its Ser-3 residue [17]. However, it is still not clear whether a similar mechanism may actually operate in differentiating neurons, since the same activating phosphorylation on LIMK can be produced by PAK kinases, operating downstream of Rac and Cdc42 [22]. Although our results do not exclude that the LIMK-Cofilin pathway could play a role downstream of TTC3, they strongly suggest that this pathway is not controlled by ROCK, at least in early differentiation. Indeed, we found that the pharmacological inhibition of ROCK in our system has no effect on the levels of phospho-Cofilin. Moreover, the overexpression of LIMK does not enhance the TTC3 overexpression phenotype, but rather rescues it, through a mechanism that requires the kinase activity and the domains which localize the protein at the Golgi or at the growth cone. Together with the previous reports showing that LIMK stimulates neuronal differentiation [21], these results suggest that, at early stages of this process, the LIMK-cofilin pathway may be much more influenced by Cdc42-Rac/PAK than by RhoA/ROCK. A second mechanism through which ROCK may affect actin polymerization is by stimulating the activity of Profilins, which in neurons are mainly represented by PIIa [41]. Our results strongly support the implication of this player. Indeed the overexpression of PIIa reverts to control values the increased neurite extension induced by TTC3 knockdown, while the knockdown of PIIa rescues both the neurite extension and the Golgi phenotypes induced by TTC3 overexpression. Moreover, this results are in line with our previous finding that, in neuronal cells, CIT-N and ROCK are in a same complex with PIIa [15].

In conclusion, besides confirming that TTC3 plays a crucial role in the early differentiation of neurons our study reveals a complex organization of the pathways by which TTC3 may regulate neurite outgrowth and Golgi organization through the actin cytoskeleton. It will be interesting to test whether the same processes and pathways are affected in experimental models of Down syndrome.

## Materials and Methods

### Ethics

For the preparation of primary hippocampal neurons, pregnant rats and embryos were sacrificed conforming to the Italian laws on animal experimentation and under the supervision of the veterinary service of our animal facility. The corresponding experimental protocols have been approved by the Italian Ministry of Health, Department of Public Veterinary Health with approval number 22/2007-A, released on 03/14/2007.

### Primary Hippocampal Neuronal Cultures

Hippocampal neuronal cultures were prepared from embryonic day 18.5 rat brains as described [60]. In brief, hippocampi were dissected and cells dissociated by trypsin (15 minutes at 37°C) and, after 5 washes in HBSS, separated with mechanical trituration using a fire polished Pasteur pipette. Neurons were plated on Poly-L-Lysine coated coverslips, placed in dishes containing CO_2_ and temperature-equilibrated Mem-Horse medium. After 4 hours incubation neurons were sufficiently attached and coverslips were placed inverted, separated by paraffin dots, onto astrocyte feeder-cell layers equilibrated in a N2 medium.

### Nucleofection

Hippocampal neurons were electroporated immediately after tissue dissociation and before plating using the rat neuron nucleofector kit (Amaxa). In brief, 500000 cells were centrifuged for 5 minutes at 1000 rpm. After that, supernatant was removed and neurons were resuspended in 100 μl of Nucleofector. Then 3 μg of DNA/5 μg of siRNA were added to the suspension. Neurons were transferred to a glass cuvette and electroporated with the Amaxa program O-003. Finally, neurons were plated on coverslips.

### Plasmids and siRNAs

The CMV-GFP-TTC3 plasmid was previously described [32]. The same cDNA was cloned in pERFP-C1 (Clontech). Expression plasmids for hemagglutinin (HA)-tagged LIMK1 and HA-ΔPDZ-LIMK1 [21] were a kind gift from Alfredo Càceres. To generate plasmids encoding HA-ΔLIM-LIMK1, HA-LIMK1-wt cDNA was amplified by polymerase chain reaction, with the following primers: forward, 5′ GCAAGCTTGCCATGATCGAACAGATCCTC 3′, reverse 5′ GCTCTAGACTCAGCGTAAT CTGGAAC 3′. PCR products were digested with HindIII and XbaI restriction enzymes and ligated to HindIII- and XbaI-digested pcDNA3 vector (Invitrogen). To generate plasmids encoding HA-kd-LIMK1, the K368M mutation [61] was introduced into the HA-LIMK1-wt plasmid using a direct mutagenesis kit (Stratagene). Profilin IIa was cloned into a pCherry-N1 plasmid from pEGFP-N1-Profilin IIa [41] using EcoRI-Xhol restriction sites. TTC3 knockdown by Rnai was performed by using the sh-mir RNAi constructs based on the pCMV-GIN(Zeo) lentiviral vector [32]. CITN knockdown by Rnai was performed by using the sh-RNAi constructs based on the pSUPER-GFP vector [15]. siRNA targeting rat Profilin IIa coding sequence (5′-UAAGCAAGGUGCCGGUGUA-3′) were purchased from Dharmacon (Thermo Scientific, Lafayette, CO).

### Antibody and Inhibitors

The following antibodies were used: rabbit polyclonal anti-GFP (Abcam, Cambridge, MA); mouse monoclonal anti-GM130 (Transduction Laboratories, BD biosciences, Franklin Lakes, NJ); mouse monoclonal anti-TAU (Chemicon); rabbit polyclonal anti-pLIMK1 (Santa Cruz, Santa Cruz, CA); rabbit polyclonal anti-Profilin IIa (kindly provided by Dr. W. Witke); rabbit polyclonal anti-cofilin phospho serine 3 (Cell signalling technology); mouse monoclonal anti-cofilin (Abcam); rabbit polyclonal anti-MLC phospho serine 9 (Cell signaling technology); mouse monoclonal anti-mlc (Cell Signaling technology); rabbit polyclonal anti-actin (Santa Cruz); mouse monoclonal anti-HA (Cell signaling technology); we obtained anti-TTC3 rabbit polyclonal antibodies by injecting rabbits with a fusion protein corresponding to aminoacids 719–1176 of TTC3. C3 cell-permeable transferase protein was obtained from Cytoskeleton (Denver, CO), dissolved in 50% glycerol and used in accordance with manufacture’s instruction. ROCK inhibitor Y-27632 (Sigma-Aldrich) was dissolved in water and used at 36,7 μM for 24h. As a control, addition of vehicle only (50% glycerol and water, respectively) was used. F-actin depolymerizing drug CytochalasinD (Calbiochem) was suspended in DMSO, added to neurons 6 hours after plating with a final concentration of 1 μM and maintained for 24 hours. The actin stabilizing drug Jasplakinolide (Calbiochem) was suspended in DMSO, added to neurons 36h after plating at a final concentration of 5 nM and maintained overnight. As a control for all the above drugs, addition of vehicle only (DMSO) was used.

### Immunofluorescence

Hippocampal neurons grown on coverslips were fixed with 4% paraformaldehyde (PFA)/PBS for 10 min. Quenching with NH4Cl 50mM/PBS was conducted to remove PFA residue for 15 minutes. Permeabilization was carried out with 0,1%TritonX-100/PBS for 3 minutes and a 5% BSA/PBS saturation (Bovine serum albumin in PBS) was left for 30 minutes over the coverslips. Following, primary antibodies (described below) were incubated for 1 hour. After three quickly washings with PBS, primary antibodies were detected with anti-rabbit or anti-mouse or anti-rat Alexa Fluor 568 or 488 secondary antibodies at 1∶1000 dilution for 30 minutes; Alexa Fluor 350 was used at 1∶250 dilution. After three quickly washings with PBS, coverslips were mounted with Mowiol on cover glasses. Images were acquired using an ApoTome system (Zeiss, Germany) or a ViCo fluorescence microscope (Nikon), or with a Leica SP5 confocal microscope.

### Live Cell Imaging

Hippocampal cultures were prepared as described above and left for 6 h before videorecording. Videorecording was performed under an Axiovert Zeiss inverted microscope equipped with an environmental chamber using a 63× oil-immersion objective. Cultures were maintained at 37°C and gassed with 5% CO2. For phase contrast imaging, exposure times were typically 5 ms, 12 frames per hour. Metamorph software (Universal Imaging) was used for acquiring images and for mounting avi format movies.

### Image Analysis

Images were analyzed with ImageJ software, or Apotome (Zeiss) and ViCo (Nikon) associated software. Differentiated cells were defined as those bearing at least one neurite longer than twice the cell body. Quantification of the percentage of differentiated cells and of cells with normal Golgi was performed in blind by two different operators. In the latter case, cells were scored as having either normal or fragmented Golgi, and the corresponding percentages are therefore complementary. The Golgi compactness index shown in Fig. 4F is an a-dimensional number calculated on serial section performed with Apotome (Zeiss). For the analysis of the circularity of Golgi apparatus, the maximum-projection from each image was derived using ImageJ software [62]. The images were then transformed in 8-bit gray-scale, 1388×1032 pixels. The Golgi labeling image threshold was set at ∼50–60 on a 0–255 black to white scale to remove background pixel from measurement. The Golgi region of interest was defined manually and the perimeter and surface was measured. The dimensionless circularity of the Golgi apparatus was computed according to the formula [40]. The colocalization analysis was performed using the ImageJ software.

### Western Blot Analysis

Neuron cortical cells were extracted with lysis buffer (1% SDS, 25mM Tris–HCl pH 6.8, protease inhibitors (Roche, Basel, Switzerland), 1 mM phenylmethylsulphonyl fluoride, 1mM Sodium Vanadate, 1mM Sodium Fluoride). Equal amounts of proteins were resolved by reducing SDS-PAGE and blotted to nitrocellulose filters, which were incubated with the indicated antibodies and developed by using the ECL system (Amersham Biosciences).

## Supporting Information

Figure S1 **Evaluation of TTC3 overexpression and knockdown in primary neurons.** **A.** Hippocampal neurons were nucleofected with GFP empty or with GFP-TTC3-expressing plasmids, plated and allowed to differentiate 36 hours in culture. The expression of endogenous and overexpressed TTC3 was then measured by western blotting with rabbit anti-TTC3 antiserum. **B.** Hippocampal neurons were nucleofected with control or TTC3-specific sh-RNA-expressing plasmids, plated and allowed to differentiate 72 hours in culture. To test the downregulation of nucleofected cells, we performed IF experiments with rabbit anti-TTC3 antiserum.
(TIF)

Figure S2 **Stage-dependent requirement of F-actin for Golgi compactness in differentiating primary hippocampal neurons.** **A–B.** Hippocampal neurons were allowed to differentiate in culture for 1 day (A) or 7 days (B) and treated with 1 μM Cytochalasin-D during the last 18 hours before fixation. The effect of treatment on actin cytoskeleton and Golgi compactness were then evaluated by staining fixed cells with Phalloidin (PHD, green in merge) and anti-GM130 antibodies (red in merge). Identical results were obtained using the drug at 2.5 μM (data not shown). Scale bars = 10 μm.
(TIF)

Movie S1 **Time lapse recording (14 hours) of exemplar primary hippocampal neuron nucleofected before plating with a GFP-expressing control plasmid.**
(AVI)

Movie S2 **Time lapse recording (14 hours) of exemplar primary hippocampal neuron nucleofected before plating with a TTC3-expressing plasmid.**
(AVI)
