# Supplementary figures and images for: The DCR Protein TTC3 Affects Differentiation and Golgi Compactness in Neurons through Specific Actin-Regulating Pathways
Source: PLoS One. 2014 Apr 2;9(4):e93721. doi: 10.1371/journal.pone.0093721 (PMC3973554; doi:10.1371/journal.pone.0093721)

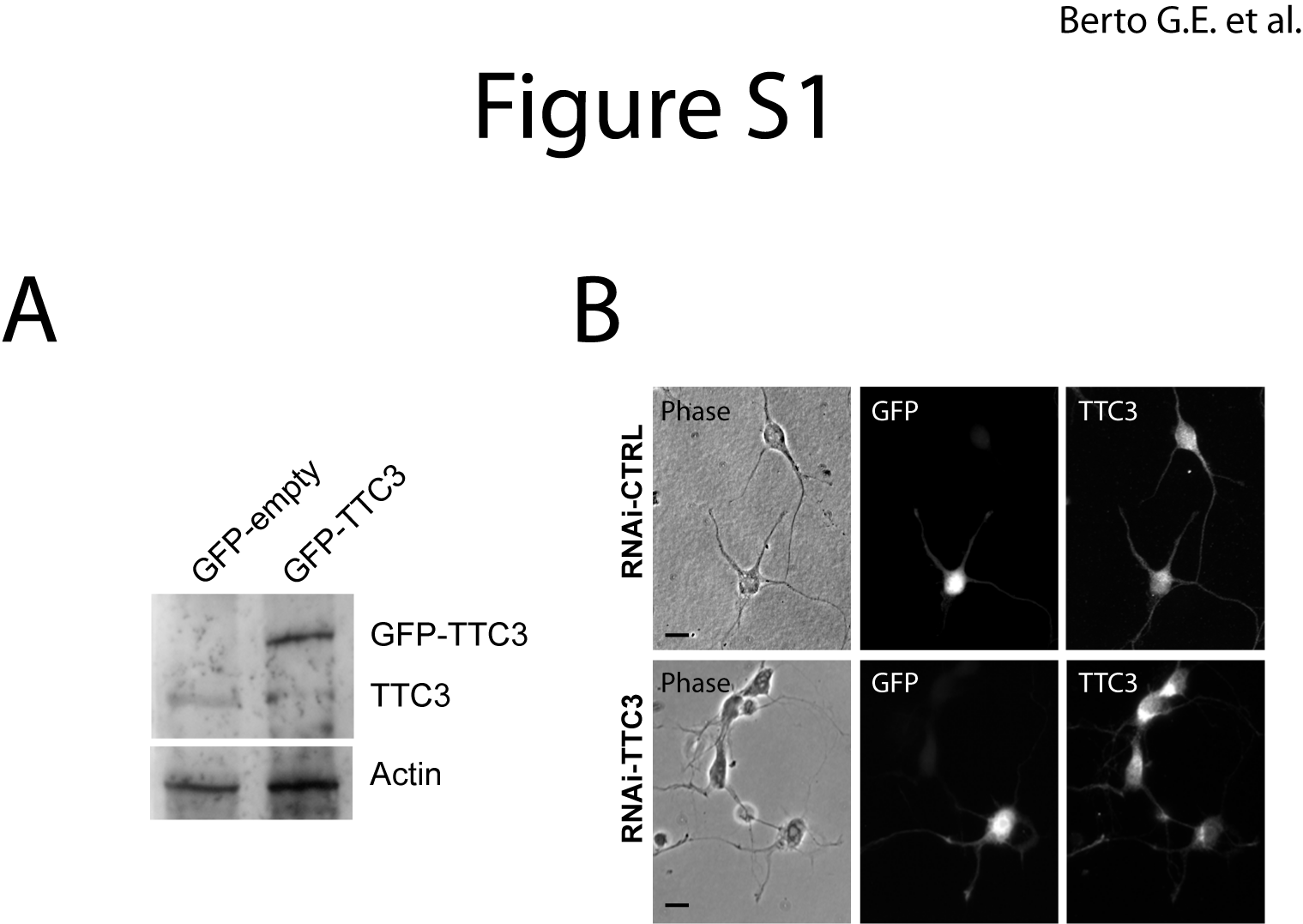

Supplement: Figure S1 — Evaluation of TTC3 overexpression and knockdown in primary neurons. A. Hippocampal neurons were nucleofected with GFP empty or with GFP-TTC3-expressing plasmids, plated and allowed to differentiate 36 hours in culture. The expression of endogenous and overexpressed TTC3 was then measured by western blotting with rabbit anti-TTC3 antiserum. B. Hippocampal neurons were nucleofected with control or TTC3-specific sh-RNA-expressing plasmids, plated and allowed to differentiate 72 hours in culture. To test the downregulation of nucleofected cells, we performed IF experiments with rabbit anti-TTC3 antiserum. (TIF) [file pone.0093721.s001.tif]

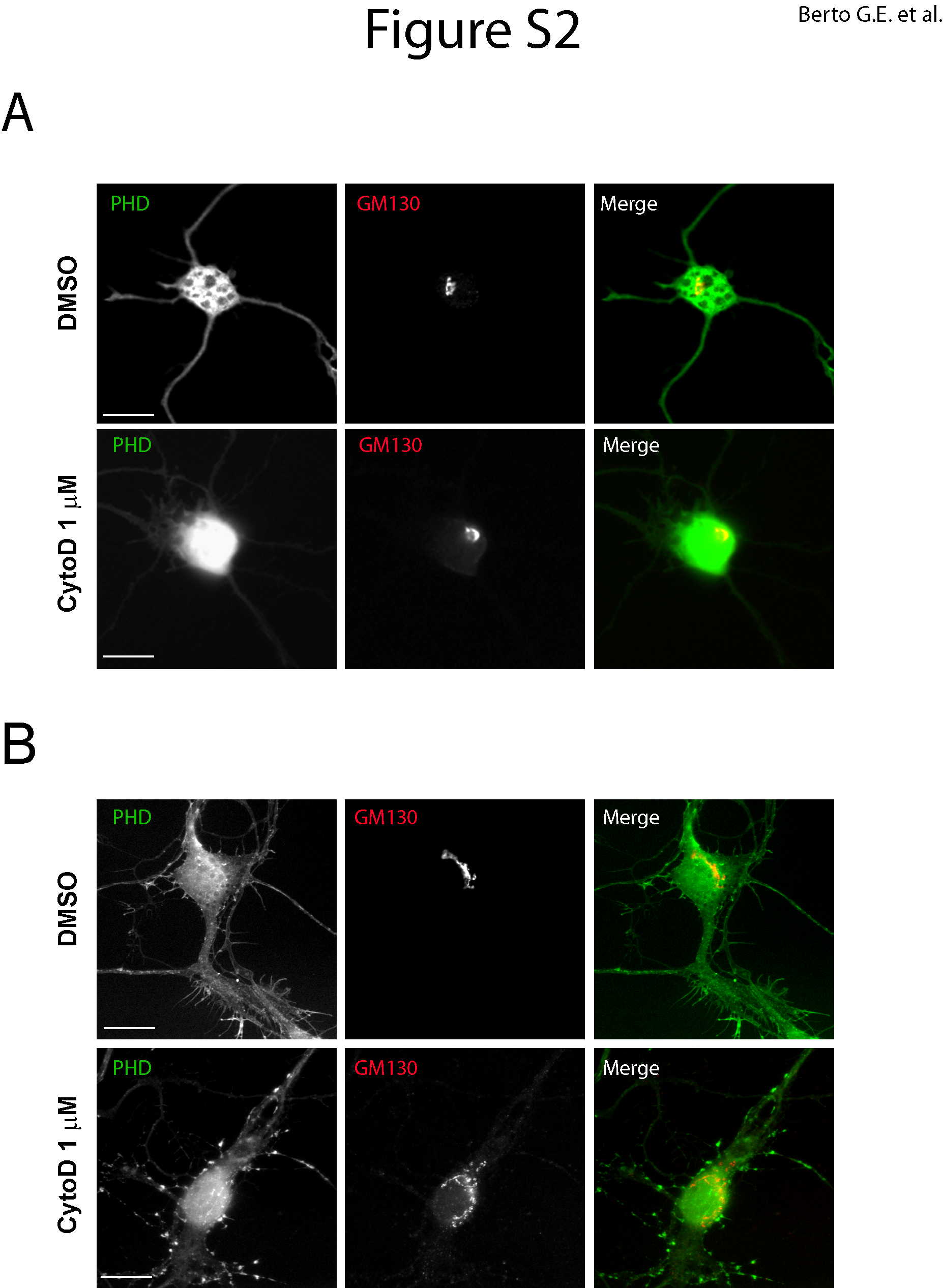

Supplement: Figure S2 — Stage-dependent requirement of F-actin for Golgi compactness in differentiating primary hippocampal neurons. A–B. Hippocampal neurons were allowed to differentiate in culture for 1 day (A) or 7 days (B) and treated with 1 μM Cytochalasin-D during the last 18 hours before fixation. The effect of treatment on actin cytoskeleton and Golgi compactness were then evaluated by staining fixed cells with Phalloidin (PHD, green in merge) and anti-GM130 antibodies (red in merge). Identical results were obtained using the drug at 2.5 μM (data not shown). Scale bars = 10 μm. (TIF) [file pone.0093721.s002.tif]
